# A Method for Automatic Surface Inspection Using a Model-Based 3D Descriptor

**DOI:** 10.3390/s17102262

**Published:** 2017-10-02

**Authors:** Carlos A. Madrigal, John W. Branch, Alejandro Restrepo, Domingo Mery

**Affiliations:** 1 Departamento de Ingeniería Electrónica, Instituto Tecnológico Metropolitano, Medellín 050013, Colombia; 2 Facultad de Minas, Universidad Nacional de Colombia, Medellín 050041, Colombia; jwbranch@unal.edu.co (J.W.B.); arestre5@unal.edu.co (A.R.); 3 Departamento de Ciencias de la Computación, Pontificia Universidad Católica de Chile, Santiago 7820436, Chile; domingo.mery@uc.cl

**Keywords:** 3D point cloud, 3D inspection, surface quality inspection, defects detection

## Abstract

Automatic visual inspection allows for the identification of surface defects in manufactured parts. Nevertheless, when defects are on a sub-millimeter scale, detection and recognition are a challenge. This is particularly true when the defect generates topological deformations that are not shown with strong contrast in the 2D image. In this paper, we present a method for recognizing surface defects in 3D point clouds. Firstly, we propose a novel 3D local descriptor called the Model Point Feature Histogram (MPFH) for defect detection. Our descriptor is inspired from earlier descriptors such as the Point Feature Histogram (PFH). To construct the MPFH descriptor, the models that best fit the local surface and their normal vectors are estimated. For each surface model, its contribution weight to the formation of the surface region is calculated and from the relative difference between models of the same region a histogram is generated representing the underlying surface changes. Secondly, through a classification stage, the points on the surface are labeled according to five types of primitives and the defect is detected. Thirdly, the connected components of primitives are projected to a plane, forming a 2D image. Finally, 2D geometrical features are extracted and by a support vector machine, the defects are recognized. The database used is composed of 3D simulated surfaces and 3D reconstructions of defects in welding, artificial teeth, indentations in materials, ceramics and 3D models of defects. The quantitative and qualitative results showed that the proposed method of description is robust to noise and the scale factor, and it is sufficiently discriminative for detecting some surface defects. The performance evaluation of the proposed method was performed for a classification task of the 3D point cloud in primitives, reporting an accuracy of 95%, which is higher than for other state-of-art descriptors. The rate of recognition of defects was close to 94%.

## 1. Introduction

Industrial inspection has to date been an exclusively human task. However, maintaining a group of people detecting surface defects accelerates visual fatigue and misinterpretations. Automatic visual inspection allows for detection of defects by image analysis. This process improves product quality, increases the production rate, avoids errors caused by subjectivity, integrates other systems into the production line, and reduces costs [1,2,3]. Industrial machine vision applications are classified into four quality categories according to the characteristics of the inspected product. These categories refer to dimensional, structural or proper assembly, superficial, and operational features [4]. An estimated 10% of failures in the manufactured parts are caused by superficial defects [5]. The surface quality inspection searches for holes, scratches, cracks, wear, finish, roughness, texture, joints, folds, discontinuities, etc. [6].

Approaches to surface defect detection are summarized in two main research directions. The first approach uses complex lighting systems to generate contrast changes in the surface defect [7]. These methods detect and recognize the defect from the measurement and characterization of pattern deformation on the surface of the object. Zhi and Johansson [8] presented a method for interpreting the deformation of the fringes based on 14 shape features without measuring the depth. Caulier et al. [9] proposed a set of eight features suited to the problem of surface inspection, combined with six characteristics that were developed for the classification of defects in interferometry images. Osten et al. [10] carried out a study of the behavior of the fringes in different defects with the aim of developing inspection systems based on knowledge. Li et al. [11] proposed an automatic inspection scheme for fabric defect detection using smart visual sensors. The system consisted of multiple smart visual sensors working independently. Martínez et al. [12] presented a machine vision system, with an easily configurable hardware-software structure, for surface quality inspection of transparent parts. Neogi et al. [6] presented a detailed review of vision-based steel surface inspection systems. Also, a comparison was made by typology of the defect, extracted features, and detection accuracy between different defect detection systems. In [13] a machine vision system able to achieve fused image acquirement and defect inspection for the textured surface with a suitable efficiency and accuracy was presented. The second direction of investigation focuses on the 3D surface reconstruction of the object, and from the 3D point cloud features are extracted to detect and classify the defect. Pernkof [14] proposed an approach for the 3D reconstruction of raw steel blocks in industrial environments using light sectioning. Ogun et al. [15] proposed a method for identifying conical defects on simple surfaces and measuring its volume automatically from the 3D reconstruction of the part. Chu and Wang [16] presented a automated vision-based system for measure the weld bead size and detect defects. Song et al. [17] proposed a method for fabric defect identification in the textile industry using three-dimensional color phase shift profilometry. A procedure to extract fundamental quality parameters to assess the quality of welds was proposed in [18]. They used a structured light system to obtain 3D reconstruction.

The works that interpret the deformation of the illumination on the defects in the 2D image present some limitations. The 2D image does not provide enough information to enable recognition between different typologies of defects. When there are variations in the scale and geometry of defects, determining the appropriate lighting system is a challenge. The geometry and texture of the object generate brightness and shadows that may be confused with a defect. From the 2D image, it is not possible to perform precise metrology of the defect. In the literature some approaches have been reported to detect and recognize defects from the 3D reconstruction of the piece. However, this is in specific domains of an application where the geometries of surfaces have few variations and only one type of defect is recognized. Because of the above, the methods of describing the defect are simple, which limits them to recognition of a set of larger defects.

The problem of recognizing defects in a 3D surface is similar to the task of recognizing objects in 3D images from partial views. This is not a simple problem, taking into account different variations that the object/surface may suffer, such as translation, rotation, scaling, noise addition, lack of information, and in some cases, non-rigid deformations. For this reason it is necessary to create robust descriptors for different variations suffered by the defect and the surface. In  [19] a method for the extraction of characteristics is proposed, called Point Signature, which creates a signature of each point using the intersection with the surface of a sphere centered at the point. A disadvantage of this method lies in the calculation of what the author called signature point, as the intersection of the sphere with the surface is not easy to calculate and in some cases it is necessary to interpolate points, which leads to a reduced accuracy of the signature point and increases the computational cost. In addition, the calculation of the reference vector is sensitive to noise.

Johnson and Hebert [20] proposed Spin Image, which is a point descriptor based on the projection of the adjacent 3D points on a tangential 2D plane. It obtains a 2D image for that point, which becomes the characteristic. A disadvantage of Spin Image is its high dependence on the resolution of the method. Some modifications have been proposed for interpolation, increasing the computational cost. In  Reference [21] a method called the Point Feature Histogram (PFH) is proposed to describe the surface of the neighborhood of a point by the difference between the normals. Although an advantage of the method is the simplicity of its calculation, a greater disadvantage lies in the construction of the histogram for each point from the calculation of the differences between normals of an entire region. Full connectivity is created, which generates a smoothing effect for small changes in the region. This also affects the processing times. In Reference [22] a modification to the PFH method improving the processing time was presented, called the Fast Point Feature Histogram (FPFH). It does not create full connectivity for the construction of the histogram, at the cost of reducing the discriminant ability of the method. In Reference [23] the Viewpoint Feature Histogram (VFH) is presented, which is a modification to the FPFH method to describe an object globally by coding its geometry and point of view.

In the recognition of surface defects of objects on a submillimeter scale, the acquisition conditions strongly affect the recognition process. Moreover, it is considered that defects are composed of abrupt changes in the surface, which generate glare and shadows in the 3D reconstruction process and cause low point density in these defective regions. Therefore, the descriptors used to represent the underlying surface must be sufficiently stable to conditions of low point density and noise in the data. The PFH, FPFH and Spin Image, by including the normals in the calculation of the descriptor, are representation methods that highly depend on the correct estimation of the normals and their support radius, which is difficult to ensure in the presence of a defect. The PFH uses the difference between all the normals in the vicinity of a keypoint. As such, in regions with small changes the normals of these points are averaged with the normals of regions with few changes, hence causing a loss of information when describing the defect region.

This paper proposes a descriptor that allows discrimination of different regions within a 3D surface. Our descriptor is based on the estimation of local models on the surface and the difference between the model’s normals in a local region.

The contributions to this paper can be summarized as follows:

- The new local 3D surface descriptor the Model Point Feature Histogram (MPFH) is presented, improving robustness and discriminant capabilities of the PFH method.The MPFH descriptor is constructed from the estimation of models and their weighting to the formation of the underlying region.
- Furthermore, a methodology for the automatic surface defects inspection is presented. For the detection of the defect, the local 3D descriptor MPFH is used in 3D reconstructed objects. From the projection 2D of the detected 3D primitives are extracted 2D features that allow to classification of the defect.

This paper is organized as follows: Section 2 provides an overview of the methodology. It emphasizes the calculation of structures, estimation of the normals, and the proposed descriptor. Section 3 describes the experimental results and, finally, the conclusions are drawn in Section 4.

## 2. System Overview

**Figure 1:**
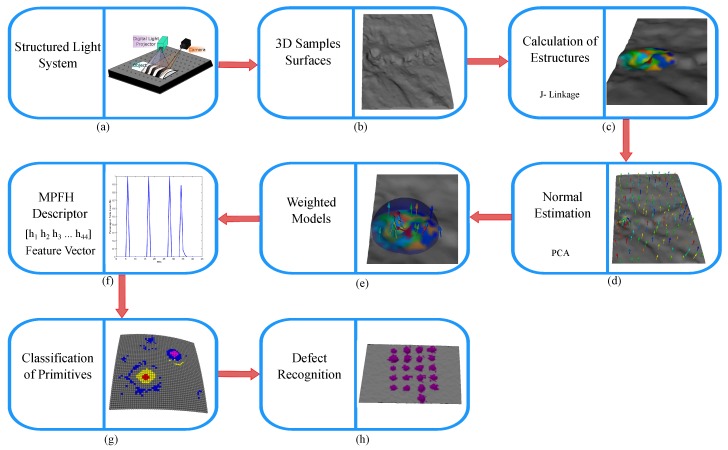
The overview of our system. (**a**) Calibration and 3D reconstruction using structured light; (**b**) representation through the point cloud; (**c**) calculation of multiple structures; (**d**) estimation of normal vectors by principal component analysis; (**e**) calculation of the contribution of each model to the formation of the region; (**f**) construction of a histogram as a 3D local descriptor; (**g**) classification of the point cloud in primitives; (**h**) surface defect recognition. MPFH: Model Points Feature Histogram.

Figure 1 illustrates the scheme of the methodology proposed to recognize surface defects. Our methodology begins with surface 3D reconstruction by projecting structured light patterns (Figure 1a). As a result, a 3D point cloud is obtained which represents the sampled 3D surface (Figure 1b), followed by the calculation of the models or planes that fit each region best using the multiple structures estimation method J-linkage [24] (Figure 1c), and then the normals of the surface models are estimated (Figure 1d). For each surface model, the model contribution weight to the formation of the surface region (Figure 1e) is calculated and from the relative difference between two models of the same region a histogram is generated representing the underlying surface changes (Figure 1f). Each point on the 3D surface is classified into one of five types of primitives, points belonging to hollow, hollow edge, crest, base crest, or flat surfaces (Figure 1g). In this work, it is considered that with this set of primitives it is possible to describe any typology of the defect. For instance, a bump defect will be composed of the base crest, crest, and possibly flat primitives; therefore regions with local geometric changes are detected on the surface. Finally, these regions are extracted 2D features to recognize the defect in a classification stage (Figure 1h).

### 2.1. Image Acquisition

**Figure 2:**
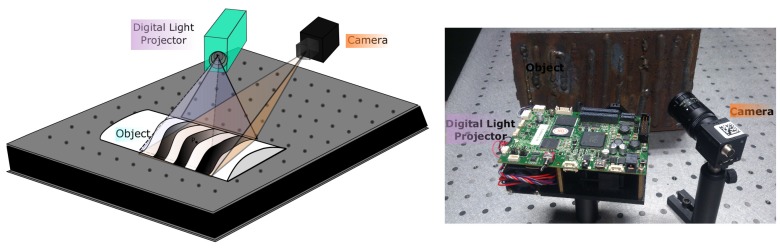
Scheme of the 3D reconstruction system.

The 3D point cloud is acquired from a 3D reconstruction system. The technique of projecting structured light patterns was used. Specifically, sinusoidal phase shift patterns were projected. A complementary metal-oxide semiconductor (CMOS) camera (Point Grey FL3-U3-88S2C-C, Richmond, BC, Canada) was used to capture the images and a digital light projector (DLP) was used to synchronously project the sinusoidal patterns on the object [25]. The acquisition scheme is shown in Figure 2. The specifications of the camera and the projector are shown in Table 1. Our 3D reconstruction system had an RMS measurement error of 0.053 mm in *x*, 0.039 mm in *y* and 0.10 mm in *z*, for a measurement area of 60 mm × 60 mm.

### 2.2. Calculation of Structures

**Figure 3:**
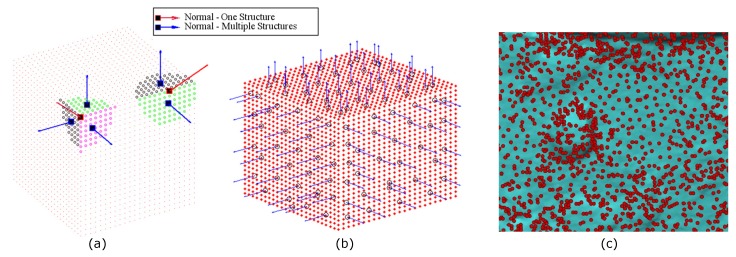
(**a**) Comparison of calculation of normals in edge and corner using the estimation of one and multiple structures; (**b**) calculation of normals for a cube using J-linkage for estimating multiple structures; (**c**) estimated model centers on a defective surface.

Superficial defects generate deformations on the object’s surface; this causes the normals and curvature in a defective region to change with respect to the neighborhood region. Therefore, if the representation method includes the normal or curvature in the construction of the descriptor, its adequate estimation influences the description of the region. Given a point cloud of the sample 3D surface called *S*, let $P_{i}\in S$ be point on the surface; the *k* closest adjacent points around $P_{i}$ are denoted by $R_{k}$. The problem of choosing *k* is known as the correct scale factor and it affects the estimation of the normal vectors. The parameter *k* is also related to the minimum defect size that our system is capable of detecting. To estimate the normal to a point $P_{i}$ on the surface, a least squares local plane is fitted to $R_{k}$ and its normal vector becomes normal to the point $P_{i}$ [26]. However, the normals of the planes that are adjusted to points $R_{k}$, with very big *k* or belonging to edges or corners, undergo a smoothing effect leading to misdirection, which influences the description of that region. The normals shown in red in Figure 3a present these effects.

A structure is defined as a set of points that adjusts to a plane. We call these structures “models” as they represent the surface changes in a simple manner. The region $R_{k}$ around the corner is composed of three structures or models with different normal vectors (Figure 3a). Thus, our methodology proposes using a method to estimate the multiple structures $M_{j}$ in a region $R_{k}$ and then calculate their normal vectors. A model is denoted by $M_{j}={\left\{p_{i}\right\}}_{i=1:\tau ,\tau \leq k}$. Moreover, it associates the normal vector of the model $M_{j}$ to each point $P_{i}\in M_{j}$, which makes it not strictly necessary to calculate the normals for each point of the surface, but rather ensures that the joining of the points that belong to the generated models is equal to the surface *S*. This is shown in Equation (1), where *n* represents the total number of models found on the surface. Figure 3b shows the normal vectors on the surface using the proposed methodology, where is possible observe the correct directing of the normals for points belonging to edges and corners. The number of $M_{j}$ models in a region $R_{k}$ depends on its topology, so that if the region has few variations, the number of found models is low compared to a region presenting many topological changes. Figure 3c represents this concept, where the red dots indicate the center of each estimated model on a defective surface.

(1) $$S=\overset{n}{\underset{j=1}{⋃}}M_{j}$$

The nature of the problem described above is comprised of a set of data that belongs to multiple structures and outliers. The challenge is to estimate the different structures in the presence of noise and geometric changes in the point cloud.

We use the approach proposed in [24], the J-linkage method, to estimate the multiple structures on a local region of points $R_{k}$, which is composed of the *k*-nearest neighbors of a point $P_{i}$. As in Random Sample Consensus (RANSAC) [27], J-linkage generates a random set of *M* hypothesis models called a minimum sampling set. Then the consensus set (CS) of each model is determined as the set of points that is at a smaller distance than a threshold ϵ for this particular model. From the CS of the *M* hypothesis models, a 1×M vector is built, representing the set of models to which $P_{i}$ belongs, where 1 in the column *j* of the vector represents the belonging of that point to the $M_{j}$ model and 0 represents that the point does not belonging to the model. This vector is called the preference set (PS) of a point $P_{i}$, which becomes a conceptual representation of this point, so that points within the same structure will have similar representations. They will be grouped together as in [24], in a conceptual space ${\left\{0,1\right\}}^{M}$. To measure this similarity, the Jaccard distance is used (Equation (2)). *A* and *B* are two PSs different to ⊘ and $d_{J}$ takes on values between 0 and 1, for identical and different sets, consecutively.

(2) $$d_{J}\left(A,B\right)=\frac{\left.A\cup B\right.-\left.A\cap B\right.}{\left.A\cup B\right.}$$

J-linkage has been used to determine the multiple flat structures in a scene for segmentation purposes from point clouds [28,29], where the difference between the functions of the flat structures is high and the amount of structures is low compared to the density of points. This difference does not arise with point clouds belonging to 3D reconstructions of surface defects, where noise can easily be an inlier of structures and generate a very large number of structures. Therefore, we first generated local regions $R_{k}$ throughout the point cloud, and then the models of each $R_{k}$ region were determined using J-linkage. As a result, the estimated models represent local changes in the surface.

A model of a plane can be represented from up to three points. However, we found experimentally that accepting models with fewer than five points increases false models due the noisy data. Increasing the number of points results in that in defective regions, where the point density is low, the models are rejected.

### 2.3. Normal Estimation

After obtaining the *M* models belonging to a neighborhood $R_{k}$ of $P_{i}$, the normal vector to the model is determined from the analysis of the eigenvalues λ and eigenvectors $\vec{v}$ of the covariance matrix *C*, as described in Equation (3).

(3) $$C=\frac{1}{\tau }\sum_{i=1}^{\tau }\left(P_{i}-\bar{P}\right)\cdot {\left(P_{i}-\bar{P}\right)}^{T}$$

The eigenvector $\vec{v_{0}}$ of the smallest eigenvalue $\lambda_{0}$ corresponds to the approximation of the normal $\vec{N}$, if the condition $\lambda_{2}\geq \lambda_{1}\geq \lambda_{0}\geq 0$ is fulfilled [30].

**Figure 4:**
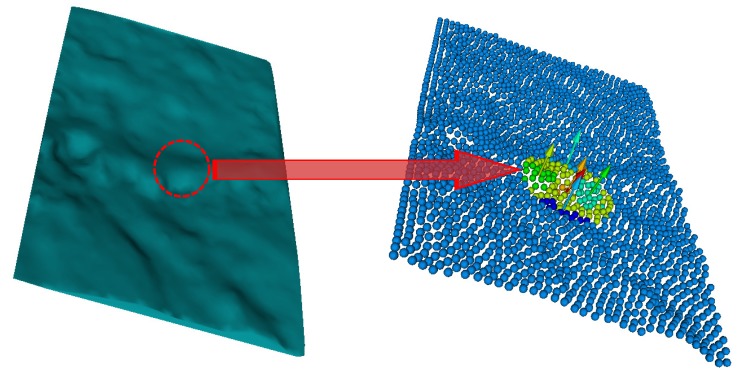
Normal estimation on hole defect by the proposed methodology.

Using models to determine the normal vectors to a surface of a region solves to a large extent the problem of the right scale factor when calculating the normals (see Section 3.2). The problem of the scale factor is associated with the selected support radii to estimate the normals. If the scale factor (*k* or *r* search) is big, the region will undergo a smoothing effect and the normal vector will not capture its details. Algorithm 1 shows the pseudo-code for determining the models and their associated normal vectors and Figure 4 shows the result of the estimation of the normal vectors on a surface sampled containing a hole defect.

**Algorithm 1:** Normals estimation
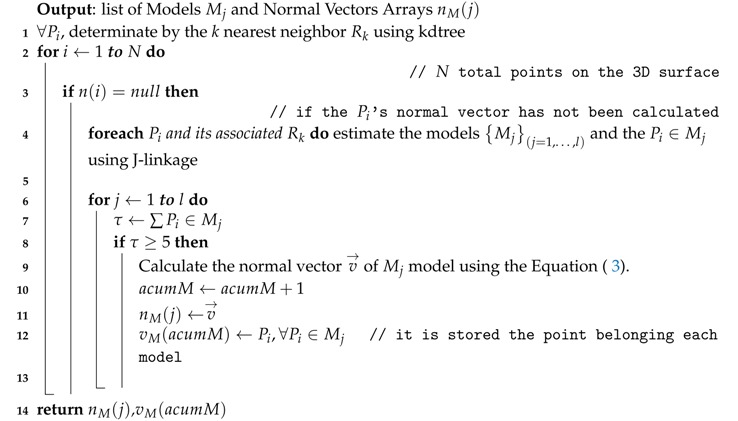


### 2.4. Model Points Feature Histogram (MPFH)

After obtaining the models and its normal vectors, it is necessary to calculate a representation for each point $P_{i}$ of the surface sampled *S*. For this, we propose calculating a histogram of characteristics similar to that proposed in [31].

The calculated feature is the relative difference between the normal vectors of each two models belonging to the region $R_{k}$ in a Darboux coordinate system. This coordinate system generates a 4D feature that is invariant to the translation and rotation, which has the advantage of reducing the number of parameters from 12 to 4 parameters. This reduction is composed of the coordinates $P\left(x,y,z\right)$ and normal vectors $N\left(x,y,z\right)$ of the two points.

The pair $\left(P,N\right)$ is known as surflet. The origin will be $P_{1}$, if Equation (4) is met, otherwise it will be $P_{2}$ and the indexes 1 y 2 in the equations must be exchanged. This is to achieve homogeneity in the choice of the origin of the coordinate system.

Let · denote the scalar product between two vectors and × the cross product of the two vectors.

(4) $$\left|N_{1}\cdot \left(P_{2}-P_{1}\right)\right|\leq \left|N_{2}\cdot \left(P_{2}-P_{1}\right)\right|$$

**Figure 5:**
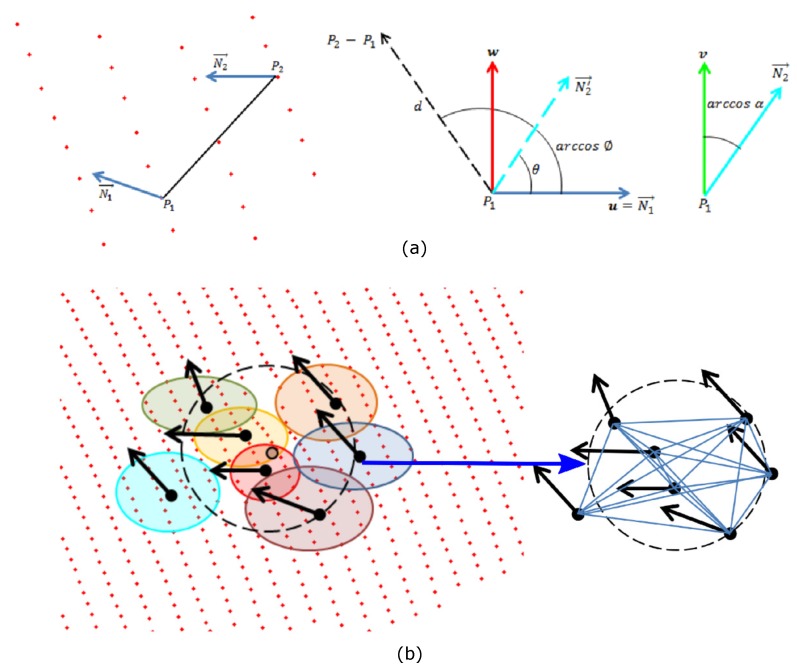
Darboux features. (**a**) Darboux coordinate system. (**b**) Procedure for the construction of the MPFH descriptor.

The axes of the coordinate system are expressed in Equations (5)–(7), and the features indicating the differences between two surflets are described in Equations (8)–(11), [31]. Figure 5 shows the relationship between the coordinate systems and the features obtained.

(5) $$u=N_{1}$$

(6) $$v=\frac{\left(P_{2}-P_{1}\right)\times u}{∥\left(P_{2}-P_{1}\right)\times u∥}$$

(7) w=u×v

(8) $$\theta =\arctan\left(w\cdot N_{1},u\cdot N_{2}\right)$$

(9) $$\alpha =v\cdot N_{2}$$

(10) $$\phi =u\cdot \frac{\left(P_{2}-P_{1}\right)}{∥\left(P_{2}-P_{1}\right)∥}$$

(11) $$d=∥\left(P_{2}-P_{1}\right)∥$$

To calculate the descriptor of a point $P_{i}$, the closest points $P_{k}$ are searched in a neighborhood $k_{2}$ which we call region $R_{k2}$. Then, the models $M_{j}$ and their centroids to which belong to the region $R_{k2}$ are determined. Hence, a vector with the set of models and another with the level of participation ρ of the model in the representation of the region $R_{k2}$ are computed. ρ represents the ratio between the number of points that form a model $M_{j}\in R_{k}$ and the totality of the points $M_{j}$, described in Equation (12). ρ ranges from (0,1]; ρ=0 is not present, because $R_{k2}\cap M_{j}\neq 0$. Figure 5 illustrates the $M_{j}$ models belonging to the $R_{k2}$ region and the difference between them.

(12) $$\rho_{i}=\frac{R_{k2}\cap M_{i}}{M_{i}}$$

The PFH method [21] uses the histogram of characteristics proposed in [31], where the features $\left(\theta ,\alpha ,\phi ,d\right)$ are found between each pair of surflets belonging to the region $R_{k2}$. It generates a set of $k_{2}\left(\frac{k_{2}-1}{2}\right)$ quadruplets of features with complete connectivity without weighting. As a result, it averages the differences between surflets and smoothes local details of the region. On the other hand, our method is based on the relationship between the normal vectors of the models belonging to the region $R_{k}$. In addition, our proposal adds the parameter ρ, which represents the participation of each model to the formation of the region. This speeds up the calculation of the representation and leads to a better capture of the geometric information of the underlying 3D surface. This also reduces the computational complexity.

**Figure 6:**
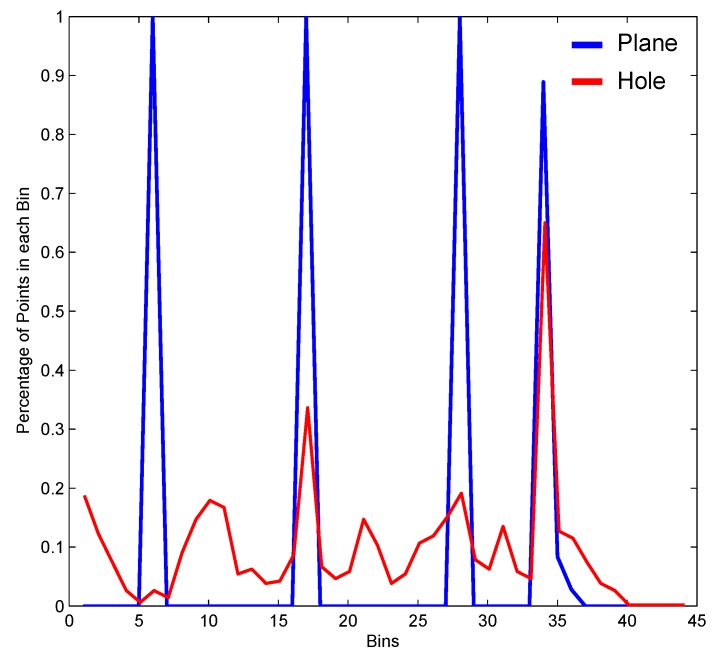
Procedure for the construction of the MPFH descriptor of a point belonging to a flat region and of a point belonging to a hole-like defect.

To calculate the MPFH representation of each point $P_{i}$, a histogram is generated from the quadruplets of the region $R_{k2}$ using the first three Darboux features and adding the weighting of the model to the formation of the region $\left(\theta ,\alpha ,\phi ,\rho \right)$. Therefore, a histogram *H* with 4 × *b* bins is generated, where the first *b* bins corresponds to the quantization of the feature ϕ, the following to α, then to θ, and finally the last corresponds to ρ. Figure 6a shows the MPFH representation for a point belonging to a flat region and Figure 6b for a point belonging to a hole-like defect, given *b* equals 11 as in the FPFH method.

### 2.5. Primitives 2D Projection

**Figure 7:**
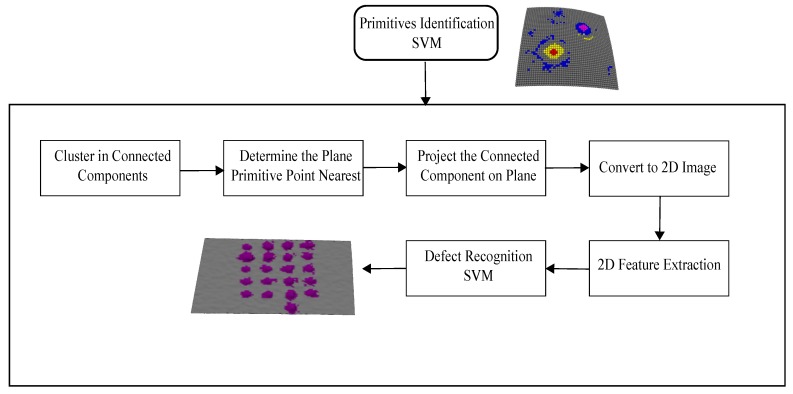
Scheme recognition. SVM: Support vector machine.

The identification of primitives allows for detection of defects on surface but not their classification. Our proposal is to project each connected component of primitives on a plane forming a 2D image. Then, 2D geometrical features are extracted for recognition of the defect. Figure 7 shows the methodological scheme proposed.

**Figure 8:**
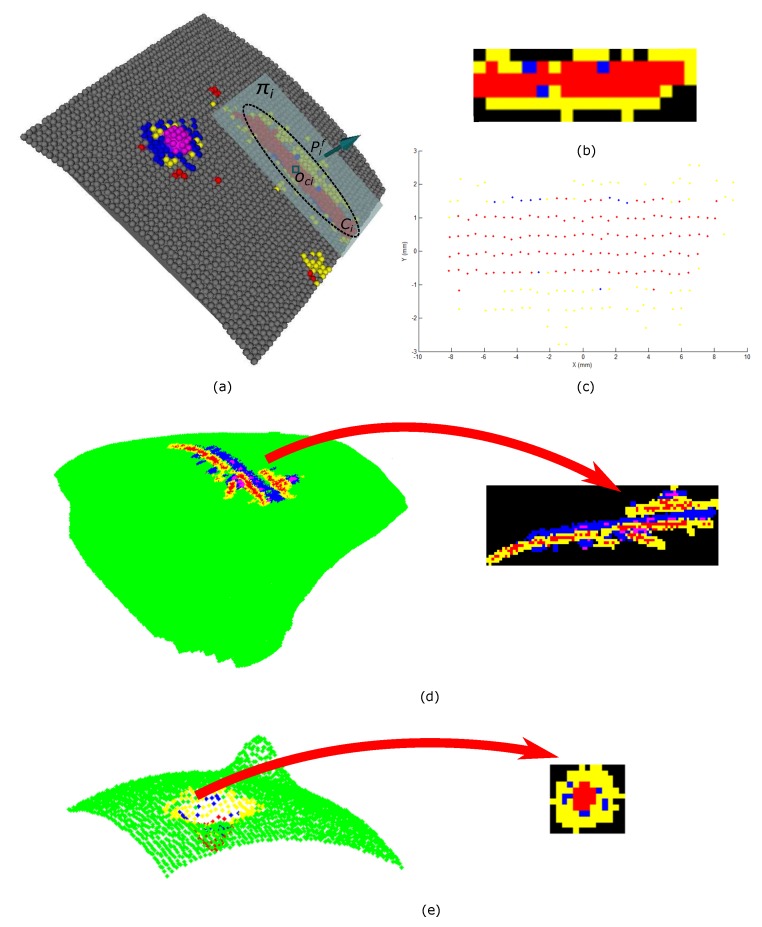
A 2D image of the defect. (**a**) Estimation of projection plane; (**b**) primitive projection; (**c**) a 2D image of primitives; (**d**) a 2D projection of a defect on an artificial tooth; (**e**) a 2D projection of a defect on a simulated surface.

First, the primitives are clustered in connected components, using Euclidean distance. The threshold distance $d_{t}$ is selected according to resolution of the point cloud. Then, for each connected component $C_{i}$, the centroid $O_{ci}$ is calculated, and with the flat primitive $P_{i}^{f}$ nearest to this, the projection plane $\Pi_{i}$ is set. All primitives $P_{c_{i}}$ are projected orthogonally to the plane $\Pi_{i}$ as described in Equations (13)–(15). Figure 8a,b illustrates the procedure.

(13) $$q=\left|O_{\mathrm{ci}}-P_{i}^{f}\right|$$

(14) $$D=\left|q\cdot N_{i}^{f}\right|$$

(15) $$P_{\mathrm{proj}}=P_{c_{i}}-D*N_{i}^{f}$$

$N_{i}^{f}$ is the normal vector associated to $P_{i}^{f}$.

A affine transformation is applied to the plane $\Pi_{i}$, so that it is parallel to the z=0 plane with normal vector $n_{z=0}$(0,0,1). Equations (16)–(18) show the calculate of axis and angle rotation, and Equation (19) obtains the matrix Euler-Rodriguez formula.

(16) $${\mathrm{Rot}}_{\mathrm{Axis}}=N_{i}^{f}\times n_{z=0}$$

(17) $$u_{ax}=\frac{{\mathrm{Rot}}_{\mathrm{Axis}}}{∥{\mathrm{Rot}}_{\mathrm{Axis}}∥}$$

(18) $${\mathrm{Rot}}_{\mathrm{Angle}}=\theta_{g}=N_{i}^{f}\cdot n_{z=0}$$

(19) $$R=\cos\theta_{g}I+\sin\theta_{g}{\left[u_{ax}\right]}_{\times }+\left(1-\cos\theta_{g}\right)u_{ax}⊗u_{ax}$$

${\left[u_{ax}\right]}_{\times }$ denotes the cross-product matrix of $u_{ax}$, ⊗ is the tensor product, and I is the identity matrix. Figure 6b illustrates the result of this procedure.

The 2D image is formed by the conversion of the plane $\Pi_{i}$ in mm to pixels. For that, we calculate the factor $s_{x}=\frac{1}{d_{x}}$$\frac{pix}{mm}$, where $d_{x}$ is the closest distance between $O_{ci}$ projected at $\Pi_{i_{z=0}}$ and other point. Then, the bounding box of $\Pi_{i_{z=0}}$ is calculated with the goal of translating it the coordinate origin. Figure 8c shows the 2D image result and Figure 8d,e shows 2D projections of different typologies of defects on the objects of experimentation.

According to [32], the characteristics of a surface imperfection are represented in the length, width, depth, height, and area of imperfection. Hence, it is possible to combine 3D and 2D descriptors to recognize an imperfection. As explained in Section 2.4, the 3D information of the curvature of the surface is embedded in the local 3D descriptor MPFH. However, it is necessary to obtain 2D geometric information that allows recognition of the defect. We proposed to group each set of primitives from its 2D projection and then extract a set of characteristics from each region of primitives belonging to the defect. Finally, in a classification stage the defect region is recognized.

The set of characteristics was chosen from a process of selection of characteristics using a Fisher discriminant [33] through the Balu toolbox Matlab [34]. The most relevant features for each type of primitives were the Hu [H1, H2, H7] moments, the Fourier descriptors [F3, F4, F5, F7 and F11], the eccentricity [Exc], and the number Euler [En].

## 3. Results

In this section, we developed two types of evaluation. First, we proved the discriminative power of the proposed 3D local descriptor compared with five methods of description commonly used. Also, invariance of descriptors to noise and support radius was addressed. Second, we tested the performance of the proposed defects recognition methodology in a database with different objects and defect typology.

### 3.1. Database

**Figure 9:**
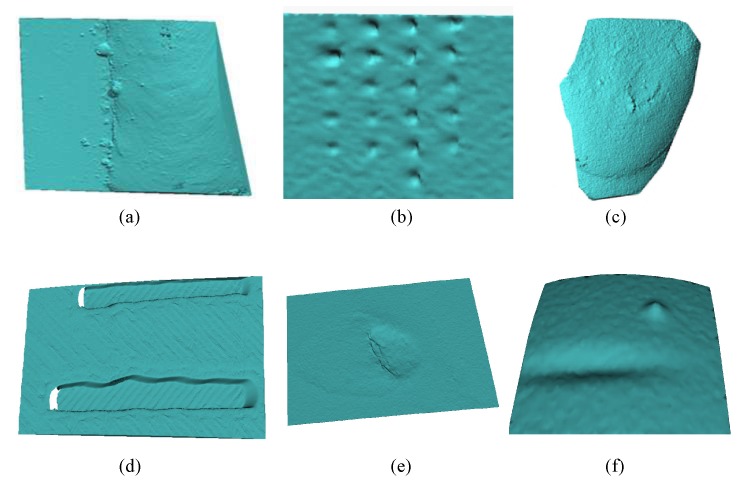
Examples from the database in: (**a**) defect in welding; (**b**) indentations in materials; (**c**) artificial teeth; (**d**) models of artificial cracks; (**e**) ceramics; (**f**) simulations.

According to our review, to date there have not been any public databases of 3D images with surface defects. Therefore, the database used in these experiments was constructed from 3D reconstructions of defects in welding, artificial teeth; indentations in materials, ceramics, models of artificial defects, and simulations. Figure 9 shows an example of the objects used in the database. Our database contains 480 three-dimensional images with more than 2000 regions with surface defects. There are four types labeled in the database: holes, bumps, cracks and without defect.

**Figure 10:**
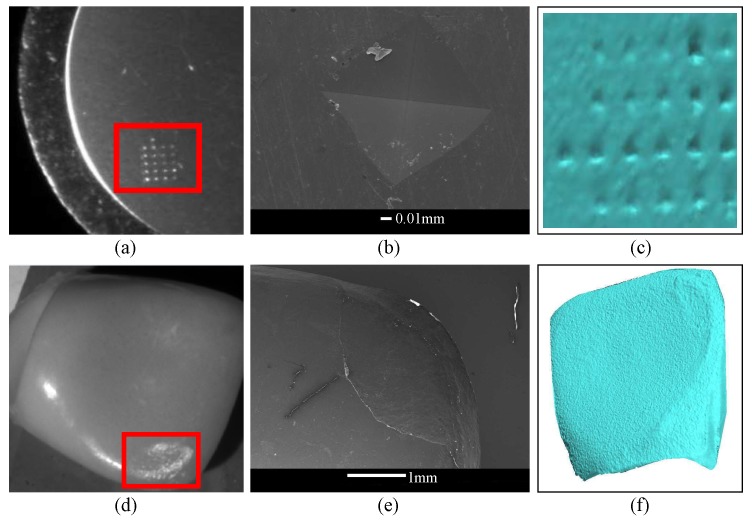
Surface defects. (**a**) A 2D image of an indentation with a test of Vickers hardness; (**b**) a scanning electron microscope (SEM) image of an indentation; (**c**) a 3D reconstruction of an indentation; (**d**) a 2D image of a detachment defect in an artificial tooth; (**e**) an SEM image of an artificial tooth; (**f**) a 3D reconstruction of an artificial tooth.

In order to understand how difficult it is to detect and recognize a micro-metric defect on an object, Figure 10a–c shows an indentation with a test of Vickers hardness on a sample of aluminum. In Figure 10d–f a material detachment defect in an artificial tooth can be observed.

### 3.2. Evaluation of the Estimation of the Normals from Multiple Structures

**Figure 11:**
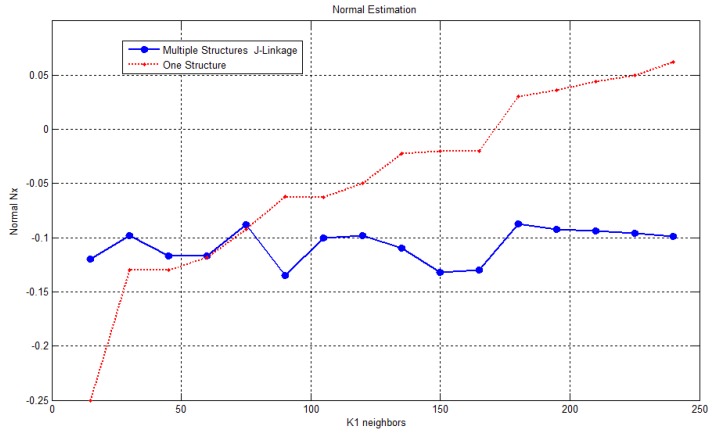
Comparison of adjustment of normal using one or multiple structures varying the number of *k*-neighbors.

In this section, we evaluate how the computation of the normal vector on a surface is affected when simple or multiple structure estimation methodologies are used. In Figure 11, the result of evaluating the change of a normal at a critical point, where abrupt geometrical changes take place, is shown. The number of k-neighbors, which supports the parameter to estimate the models, was varied between 15 and 250 points. As Figure 11 shows, the scaling factor does not significantly influence the normal vectors calculated by estimating multiple structures. In contrast to using a unique structure, the normal vector in that region changes with the variation of support radius. This means that through the proposed methodology, better-oriented normal vectors at critical points such as edges and corners are obtained. Consequently, this will increase the discriminating capabilities of our descriptor.

### 3.3. Robustness to Noise in the Estimation of the Normal Vectors

In order to evaluate the robustness to noise in the estimation of models for calculating the normal vectors, in Table 2 we show a comparison of the results of calculating the normal vectors using the adjustment of multiple- structure J-linkage versus the adjustment of a single structure on a cube. In a simple structure like a cube that has three types of regions (planes, edges, and corners), normal estimation methods based on a single structure fail at edges and corners. The defects, being more complex structures, require greater precision in the estimation of the normal, which can be achieved if the estimation of normal multiples is used. The regions chosen for the evaluation were: points in planes, near edges, and on corners. Gaussian noise with a standard deviation of 5% is also added. The calculation of the normal vectors is compared with the theoretical normal vector. The spaces in the “One Structure” column are due to the fact that, regardless of the topological complexity of a region, the methods based on the estimation of unique structures will always find only one normal vector. However, for example for a cube-type region, there are three theoretical normal vectors which match the results using multiple structures. The mean square error between the calculation of the normal vectors using the estimation of multiple structures with respect to the normal theoretical vector was 0.00854, whereas with a single structure this value was 0.01117. In summary, these results show that is more stable to calculate the normal vectors using the estimation of multiple structures versus single structure, even with the presence of noise.

### 3.4. Evaluation of the Discriminating Properties

In order to evaluate the discriminating capabilities of the MPFH method, it is compared with the PFH and FPFH descriptor with different *k* neighborhood support. The measure of similarity used for the histogram was the intersection between the descriptors for seven different regions: the point in a plane, cylinder, sphere, a point on the vertices of a triangular pyramid, square pyramid, pentagonal pyramid, and hexagonal pyramid. When the number of sides of the polygon of the base of the pyramid increases, the curvature of the vertex is close to the curvature of a sphere. These regions were chosen because they present a small change between them in the curvature.

**Figure 12:**
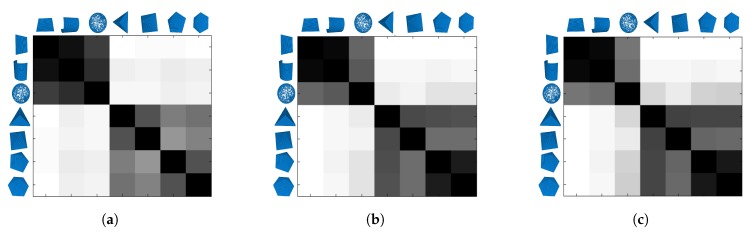

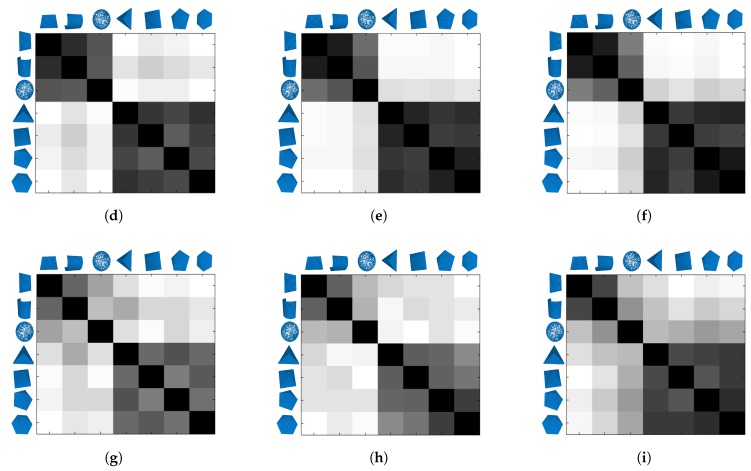
Measurement of distances between feature vectors of points in different regions and with different $k_{1}$ and $k_{2}$ neighborhood support. (**a**) PFH con $k_{1}=15$, $k_{2}=45$; (**b**) PFH $k_{1}=30$, $k_{2}=60$; (**c**) PFH $k_{1}=45$, $k_{2}=75$; (**d**) FPFH $k_{1}=15$, $k_{2}=45$; (**e**) FPFH $k_{1}=30$, $k_{2}=60$; (**f**) FPFH $k_{1}=45$, $k_{2}=75$; (**g**) MPFH $k_{1}=15$, $k_{2}=45$; (**g**) MPFH $k_{1}=30$, $k_{2}=60$ and (**i**) MPFH $k_{1}=45$, $k_{2}=75$.

Figure 12 presents a similarity matrix. The intensity level represents the quantification of similarity between objects row and column. The rows correspond to the similarity measure for the PFH, FPFH, and MPFH descriptors consecutively, and the columns represent the similarity with different $k_{1}$ and $k_{2}$ neighborhood support. An ideal similarity matrix for a classification process would have a diagonal with black and the rest white.

It can be seen in the gray levels from Figure 12g–i that there is a lower probability of confusion of the MPFH method between different classes with respect to the PFH (Figure 12a–c) and FPFH (Figure 12d–f). These results suggest that the description made by the MPFH method facilitates a correct classification of the underlying region.

### 3.5. Comparison with Some 3D Local Descriptors

In this section, the descriptor MPFH proposed is compared with some of the descriptors more often used in the literature like PFH, FPFH, spin image, radius-based surface (RSD), and signature of histograms of orientations (SHOT). From the database we carefully selected 2800 points belonging to different 3D surfaces regions. These points were categorized into five classes: hollow, crest, edge hollow, base crest and planar surfaces. Descriptors were evaluated in a classification task varying Gaussian noise, adding 0%, 5% and 10%.

**Figure 13:**
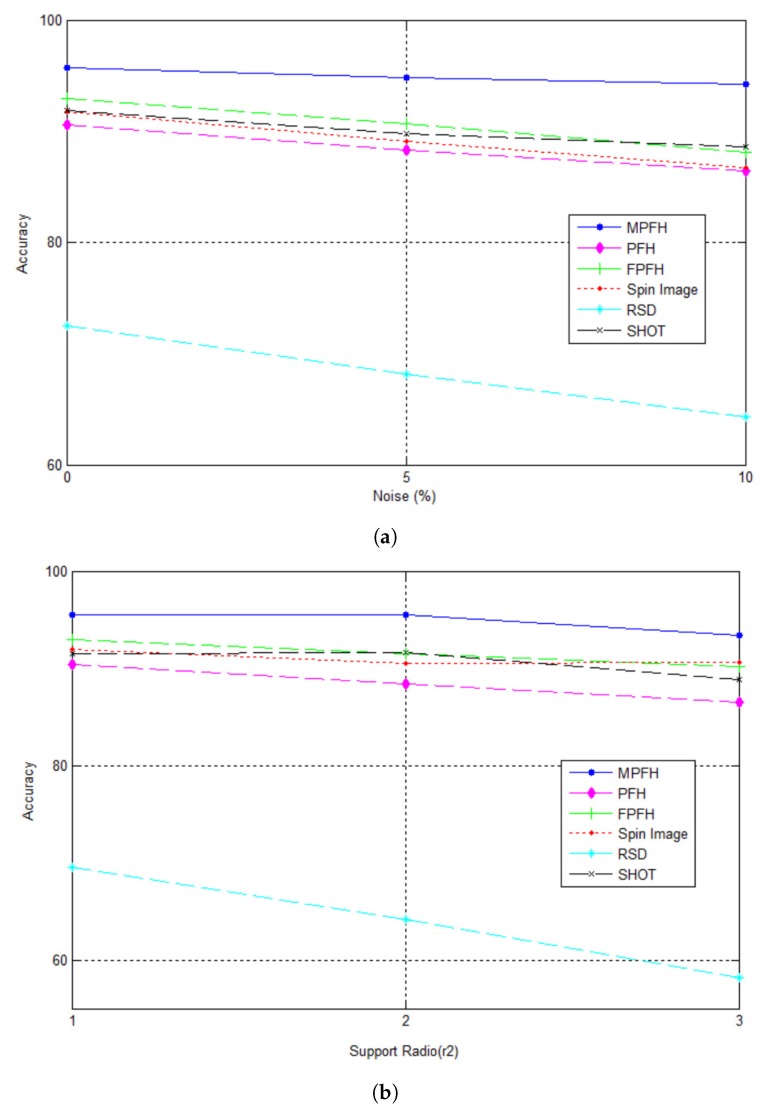
MPFH Descriptor. (**a**) Comparing different descriptors adding Gaussian noise; (**b**) comparison of different descriptors varying the $r_{2}$ support radius.

Figure 13a compares the accuracy obtained between the descriptors. It can be seen that the accuracy of the descriptor MPFH is higher, even with the addition of noise, and although accuracy decreases with increasing the percentage of noise, it provides less of a slope than the PFH, FPFH, spin image and the radius-based surface descriptor (RSD).

In Figure 13b, the classification results varying the $r_{2}$ support radius for the calculation of the descriptors are displayed. We can also observe that while decreasing the classification accuracy with the variation in radius $r_{2}$ support, our method still has the greatest accuracy between descriptors. In Figure 13b, the qualitative results taking the 3D sampled surface of the artificial tooth, welding defect, and simulated defects models with added noise as test data are shown.

### 3.6. Performance Evaluation for Defects Recognition

This section evaluates the full methodology of surface defect recognition proposed in this paper. The set of training and test images consisted of 2160 regions labeled as holes, bumps, cracks and without defect.

**Figure 14:**
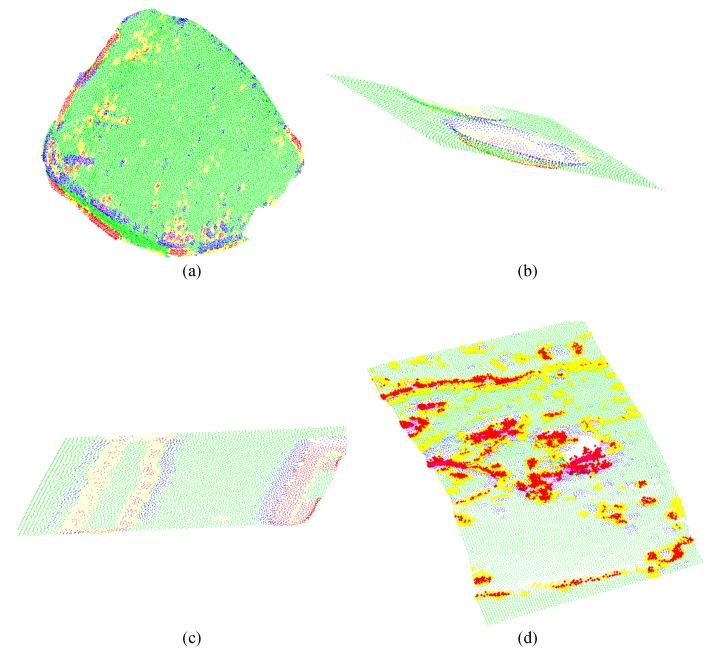
Classification of primitives on different objects. (**a**) artificial teeth; (**b**) ceramics; (**c**) models of artificial defects; (**d**) defects in welding.

In the detection stage, the points of the surface are classified into five primitives, the points belonging to a hollow (red), hollow edge (yellow), crest (magenta), base crest (blue), and flat (green) surfaces. The capacity of the local descriptor to represent and classify the regions of the surface can be seen in Figure 14, . However, it is also observed that misclassified primitives appear, which will affect the classification process.

The recognition stage was evaluated with the classification techniques: k-nearest neighbors (knn), linear discriminant analysis (LDA), quadratic discriminant analysis (QDA), multilayer perceptron, support vector machine (SVM) with radial basis function kernel (rbf), SVM with polynomial kernel function (poly), and SVM with a quadratic kernel (quad). In the evaluation process, we used 10-fold cross-validation. Table 3 shows the performance of each classifier. The performance was measured using the accuracy factor (Equation (20)).

(20) $$AverageAccuracy=\frac{\sum_{i=1}^{l}\frac{tp_{i}+tn_{i}}{tp_{i}+fn_{i}+fp_{i}+tn_{i}}}{l}$$

The parameters ($tp_{i}$) are the true positive for class $C_{i}$, the elements ($fp_{i}$) are false positives, ($fn_{i}$) are false negatives, and finally, the the parameters ($tn_{i}$) are the true negatives. The factor *l* is the class number.

**Figure 15:**
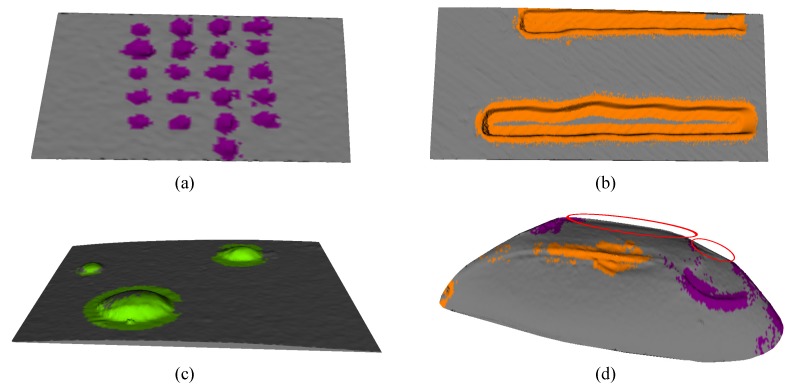
Qualitative results for the recognition of defects in (**a**) Indentation with a test of Vickers hardness; (**b**) Models of artificial defects; (**c**) Welding; (**d**) Artificial teeth. The orange region represents crack-type defects, the green region represents bumps-type defects, the purple region represents hole-type defects, and the gray color represents the regions without defects.

Figure 15 shows the result of the classification for different images in the database. It can be seen from images that the recognition stage is able to correctly classify the defect. However, can be seen that in defective regions close to the edges of the object, our method erroneously classifies the region as without defect (Figure 15d). The misclassification could be attributed to the very low density of points in this regions, which causes a misclassification in primitives, affecting the recognition.

The average processing time of the automated inspection system is composed of the acquisition step, which is delayed 0.3 s, the processing and description step with 1.1 s for a region with 1817 points and three defects, and the classification step with 0.2 s. According to the above, the automated inspection system could detect defects on a surface in 1.6 s.

## 4. Conclusions

In this paper, we have presented a new local 3D surface descriptor that improves the robustness and discriminating capabilities of the PFH method. We have demonstrated its applicability for surface quality inspection in the detection of defects on different objects. The proposed MPFH method was evaluated and compared to some of the most used state-of-the-art descriptors under a classification task. The obtained results show that our method has higher discriminating capabilities, because we tried to capture the geometric information of the underlying 3D surface more precisely by estimating the normals from the models that are adjusted to the surface and including these in the construction of the descriptor. From the identification of primitives, we propose a method of description that projects each connected component of primitives on a plane forming the 2D image. Then, 2D geometric features are extracted to recognize the defect. With this method, three types of defects (holes, bumps, and cracks), with an accuracy of 94.17%, are recognized using an SVM. In the MPFH descriptor calculation, the models that best fit the surface are estimated through an estimation technique of multiple structures or models. In this paper the models are flat surfaces, however, we believe that if models can be fitted to polynomial surfaces or splines, this would help to estimate the normal vectors more correctly and the local region would be more accurately represented.

## Figures and Tables

**Table 1:** Camera and digital light projector specifications.

Device	Specifications
Camera	Point Grey FL3-U3-88S2C-C
Sensor size	1.55 μm × 1.55 μm
Image resolution	4096×2160 pixels
Lenses	Edmund Optics *f*8.5 mm
Digital light projector (DLP)	DLP LightCrafter 4500
DLP resolution	912×1140 pixels
Synchronization circuit	freescale FRDM-K20D50M
Software	Visual C++ + OpenCV3.0 + PCL1.8

**Table 2:** Normal vectors in corners, edges, and planes of a cube, using single (standard methods) and multiple structures (method used).

Region	Theoretical Normal Vector	One-Structure Theoretical	Multiple-Structure J-linkage
	$N_{x}$/$N_{y}$/$N_{z}$	(Standard Methods) $N_{x}$/$N_{y}$/$N_{z}$	Theoretical (Method Used) $N_{x}$/$N_{y}$/$N_{z}$
Corner	0.0/−1.0/0.0	−0.5350/−0.5350/0.6538	0.0/−1.0/0.0
	−1.0/0.0/0.0	—	−1.0/0.0/0.0
	0.0/0.0/1.0	—	0.0/0.0/1.0
Corner 5% noise	0.0/−1.0/0.0	−0.5438/−0.5888/0.5979	−0.0010/−0.9997/0.0215
	−1.0/0.0/0.0	—	−0.9993/0.0234/0.02866
	0.0/0.0/1.0	—	0.0711/0.0478/0.9963
Edge	0.0/0.0/1.0	0.0/−0.7071/0.7071	0.0/0.0/1.0
	0.0/−1.0/0.0	—	0.0/−1.0/0.0
Edge 5% noise	0.0/0.0/1.0	0.0027/−0.5286/0.8488	0.0765/−0.0416/0.9962
	0.0/−1.0/0.0	—	0.0159/−0.9953/−0.0952
Plane	0.0/0.0/1.0	0.0/0.0/1.0	0.0/0.0/1.0
Plane 5% noise	0.0/0.0/1.0	0.0191/−0.0116/0.9998	0.01271/−0.0114/0.9998

**Table 3:** Results of classification of surface defects. LDA: linear discriminant analysis; knn: k-nearest neighbors; QDA: quadratic discriminant analysis; SVM (rbf): SVM with radial basis function kernel; SVM (poly): SVM with polynomial kernel function; SVM (quad): SVM with a quadratic kernel.

Classifier	knn (k = 10)	LDA	QDA	Multilayer Perceptron	SVM (rbf)	SVM (poly)	SVM (quad)
Accuracy	90.09%	86.22%	66.89%	93.95%	94.17%	87.72%	93.05%
